# The Effect of Cannabis Use on Depression

**DOI:** 10.7759/cureus.51803

**Published:** 2024-01-07

**Authors:** Jhenelle Hutchinson, Saveen Sall, Lee Stevens

**Affiliations:** 1 Department of Psychiatry and Behavioral Medicine, Windsor University School of Medicine, Cayon, KNA; 2 Department of Psychiatry and Behavioral Medicine, Louisiana State University Health Shreveport, Shreveport, USA

**Keywords:** emerging adults' cannabis use, endocannabinoid system, cannabis use disorder, cannabis use, major depressive disorder (mdd)

## Abstract

This article presents the case of a 21-year-old female with a psychiatric history of depression and a history of chronic cannabis use who presented to the emergency department after overdosing on ondansetron and was urine test positive for marijuana (tetrahydrocannabinol (THC)). The patient was later transferred to a psychiatric unit for further evaluation, and after six days of hospitalization and cessation of marijuana, the patient demonstrated gradual improvement in her mental status examination and was deemed fit for discharge with follow-up instructions. This case illustrates the effect of cannabis use and cannabis use disorder on those with major depressive disorder (MDD), the component of cannabis that worsens the symptoms of depression, the role of the endocannabinoid system (ECS) in depression, and available treatments.

## Introduction

Major depressive disorder (MDD) can be a crippling disease that is described by depressed mood, diminished interests, impaired cognitive function, and disturbed sleep or appetite [1]. MDD is twice as prevalent in women and is often associated with changes in the cognitive control network in the brain [1].

Cannabis is one of the most commonly used addictive drugs, and its use is prevalent among young people [2]. Chronic cannabis use is termed as cannabis use disorder [3]. According to the Diagnostic Statistical Manual Fifth Edition (DSM-5), cannabis use disorder is defined as follows [3]: “A problematic pattern of cannabis use leading to clinically significant impairment or distress, as manifested by at least two of the following, occurring within 12 months. Cannabis is often taken in larger amounts or over a longer period than was intended. There is a persistent desire or unsuccessful efforts to cut down or control cannabis use. A great deal of time is spent in activities necessary to obtain cannabis, use cannabis, or recover from its effects. There is a craving or a strong desire or urge to use cannabis. Recurrent cannabis use results in failure to fulfill role obligations at work, school, or home. Continued cannabis use despite having persistent or recurrent social or interpersonal problems caused or exacerbated by the effects of cannabis. Important social, occupational, or recreational activities are given up or reduced because of cannabis use. There is recurrent cannabis use in situations in which it is physically hazardous. Cannabis use continues despite knowledge of having a persistent or recurrent physical or psychological problem that is likely to have been caused or exacerbated by cannabis. Tolerance, as defined by either: (1) a need for markedly increased cannabis to achieve intoxication or desired effect or (2) a markedly diminished effect with continued use of the same amount of the substance. Withdrawal, as manifested by either (1) the characteristic withdrawal syndrome for cannabis or (2) cannabis is taken to relieve or avoid withdrawal symptoms. Severity is graded as Mild, Moderate, or Severe, depending on whether 2 or 3, 4 or 5 or 6+ of the above criteria are present."

This case report presents a 21-year-old female with a history of depression and chronic cannabis use admitted to the emergency department after overdosing on 10-20 4 mg tablets of ondansetron. This case emphasized the effect of cannabis use disorder on those with MDD, the component of cannabis that worsened the symptoms of MDD, the role of the endocannabinoid system (ECS) in depression, and available treatments.

## Case presentation

A 21-year-old female with a diagnosed history of depression presented to the emergency room after an overdose of 10-20 4mg tablets of ondansetron, and her mother called Emergency Medical Services (EMS). The patient had no previous history of suicidal attempts or previous psychiatric hospitalization. The urine test was positive for marijuana (THC), and the patient admitted to nightly usage of one to two joints since the age of 19.

On day one of admission to the emergency department, the patient denied suicidal gestures. The vital signs and review of systems were unremarkable. When initially interviewed, the patient was withdrawn and would not disclose relevant information. The patient answered “I do not know” to every question except when asked about her last menstrual period. The patient did not answer "no" when asked whether she took anything other than ondansetron 4 mg tablets. Thereafter, the patient opened up slightly to the technician who gathered that, apparently, her eight-day-old baby died last week, which seemed to be the source of the current event. The patient stated that she was not able to get any details of her baby’s cause of death. Blood work and urine tests were drawn, and the results were unremarkable except for positive urine marijuana (THC). The patient was discharged from the emergency department and transferred to a psychiatric unit with a clinical impression of depression with suicidal ideation.

On day two in the psychiatric unit, the patient was seen and evaluated and stated the following, “Yesterday, my mom thought I tried to kill myself because she saw an empty medication pack. I just took two 4 mg tablets of ondansetron and the rest of the packet was empty. I took it because I was not able to eat for two days. I was not trying to kill myself. I have been feeling depressed for the last six months. My one-week-old son passed away from natural causes back in February. It has been hard to cope and it is not easy. I don’t have anyone for support or who I can talk to when I am down.” The patient also stated that her sleep is poor (four-five hours), appetite is “fine”, and energy is “poor”, and she endorsed thoughts of guilt by stating, “I think about everything that happened but I know it’s not my fault.” The patient then denied suicidal ideation, homicidal ideation, and paranoia. The patient also denied previous suicidal attempts or psychiatric hospitalization. She however admitted to marijuana (THC) usage of one to two joints nightly since age 19. The patient's vital signs and review of systems were unremarkable. Table 1 illustrates her mental status examination on day two.

**Table 1:** Mental status examination at day two

Mental status examination	Results
Appearance	Casually dressed (hospital attire) and slumped posture
Attitude	Cooperative, calm, and withdrawn
Activity/motor behavior	No adventitious movements and limited eye contact
Speech	Slow and decreased rate
Language	Yes, able to name objects
Fund of knowledge	Yes, awareness of current events
Mood	Sad and depressed
Affect	Sad and tearful
Thought process	Absent: delusions; absent: hallucinations
Suicidal ideation	None
Homicidal ideation	None
Attention	Impaired
Concentration	Impaired
Sensorium-orientation	Oriented x3
Memory/cognition	Recent intact and remote intact
Insight	Poor
Judgment	Poor

After patient evaluation, she was assessed for MDD and cannabis use disorder and started on sertraline 25 mg PO qAM. She was counseled on substance use (marijuana, THC) and was provided with substance use resources. The patient was also enrolled in a group therapy and placed on appropriate safety precautions.

The patient's mother then provided the following history. The mother stated that the patient was doing well until two days ago; she was not acting like herself and became sad. The mother stated that the patient had been sad and down periodically since the passing of her child and close aunt, which occurred three weeks ago. The mother stated that the patient was trying to harm herself, and several empty packages of ondansetron 4 mg tablets were found. The mother stated that the patient was prescribed escitalopram 10 mg PO QD tablets by her doctor in the past, but she did not take the medication for the long term because "it did not made her feel right.”

On day six of discharge, the patient's vital signs, labs, and review of systems were unremarkable. The patient's medication regimen was well tolerated and effective at controlling systems. The patient was compliant with the medication regimen and did not experience side effects. The patient attended treatment team meetings routinely and was cooperative; the patient got along well with peers and unit staff. The treatment team determined that the patient was no longer a danger to self, danger to others, or gravely disabled and that the patient had achieved the maximum benefit of the hospitalization. Table 2 illustrates the patient's mental status examination at discharge.

**Table 2:** Mental status examination at day six

Mental status examination	Results
Appearance	Casually dressed, adequately groomed, and no apparent distress
Attitude	Cooperative, calm, and engaged
Activity/motor behavior	No adventitious movements and appropriate eye contact
Speech	Regular rate and regular volume
Fund of knowledge	Yes, vocabulary
Mood	Happy
Affect	Congruent and other (brighter, improved today)
Thought process	Linear and goal-directed
Thought content	Absent: delusions; ansent: hallucinations
Suicidal ideation	None
Homicidal ideation	None
Attention	Intact
Concentration	Intact
Sensorium-orientation	Awake, alert, and oriented x3
Memory/cognition	Recent intact and remote intact
Insight	Fair
Judgment	Fair
Status at discharge	Cognitive and behavioral status at discharge is stable.
Overall status at discharge	Patient is progressing back to baseline.

The patient's discharge plan included a referral to a grief support group within seven days post-discharge. Patient discharge medications were sertraline 50 mg QD and trazodone 100 mg QHS.

## Discussion

This 21-year-old female had a six-month history of depression that started after the passing of her one-week-old son; her symptoms intensified after the death of a close aunt three weeks ago. During this time, the patient's depression was untreated because she did not like how the prescribed medication (escitalopram 10 mg tablets) made her feel. The patient's mother stated that over the past six months, the patient's mood fluctuated, but for the past two days, her mood changed for the worse and she was not acting like herself and became visibly sad. The patient's mother found her with 10-20 empty packages of ondansetron 4 mg tablets and called EMS. Throughout this time, the patient was using one to two joints of marijuana (THC) nightly since the age of 19.

The patient spent a total of six days hospitalized, and day one was spent in the emergency department. On day one in the emergency department, the patient was withdrawn and displayed poor insight and judgment, poor sleep, and low energy, and the urine test was found positive for marijuana (THC). She was transferred to the psychiatric unit, and on day two, the patient was less withdrawn with the cessation of marijuana (THC) usage; her insight and judgment remained poor. The patient displayed slow speech, sad and depressed mood, sad and tearful affect, and impaired attention and concentration. She was given substance use counseling and was provided with substance use resources; the patient was enrolled in group therapy and placed on safety precautions; she took sertraline 50 mg QD tablets, which she tolerated. On days three to five, the patient continued adherence to the above routine, and on day six, the patient was deemed fit for discharge; she has not had marijuana (THC) since admission, and she progressed toward baseline. Her mental status on day six indicated an adequately groomed appearance, regular rate speech, happy mood, brighter and improved affect, intact concentration, and fair insight and judgment. The patient was discharged with a discharge plan of referral to a grief support group within seven days and prescription of sertraline 50 mg QD and trazodone 100 mg QHS.

Further discussion

The Role of the ECS in Depression

The CB1 receptor agonist, THC, has been used for several years by humans in the preparation of *Cannabis sativa*, which helped to support the hypothesis stating there is a relationship between cannabis use and depression. Individuals who use cannabis often cite that the elevation of mood is the driving force behind their choice [4]. However, multiple clinical trials done in the 1970s aimed to determine the antidepressant efficacy of THC, which illustrated that it did not improve symptoms of depression and produced undesirable adverse effects. There is a similar hypothesis stating that individuals who are depressed self-administer cannabis for mood elevation; however, available data did not support this finding. In a recent study, this prediction was not justified and instead showed that depressed subjects encountered more depression, aggression, and sadness when intoxicated with cannabis than when they were not intoxicated [4].

The details described above led to the hypothesis that dysregulation of the ECS resulted in depression. This hypothesis is supported by the adverse events' profile in humans of the CB1 receptor antagonist rimonabant, which showed a small but significant increased change for the development or exacerbation of depression [4]. The change in depression or mood changed with depressive symptoms and increased when patients with preexisting depressive illness were not excluded from rimonabant treatment. These data indicated that endogenous activation of CB1 receptors served as a buffer against depression and its elimination or reduction in susceptible individuals can result in depressive symptoms [4].

The ECS plays a vital neurodevelopmental role. The main brain cannabinoid receptor CB1 has a greater binding affinity in adolescence than in adulthood; it gets activated by delta-9-tetrahydrocannabinol THC, the dominant psychoactive part of marijuana. This binding regulates the reward system within the brain ventral tegmental area (VTA), which is responsible for the increased release of dopamine. Recurrent CB1 binding from exogenous cannabis (THC) exposure causes downregulation of the ECS in the limbic region, such as the hippocampus [5].

The endogenous ECS is associated with mood and executive function deficits due to the accumulation of CB1 receptors in the prefrontal and limbic regions. The frontal and limbic regions of the brain neuroanatomy change from adolescence to young adulthood, and similarly the ECS changes. As a result, the use of cannabis leads to a rise in depression, executive dysfunction, anxiety, and increased impulsiveness, particularly in adolescents and emerging adults [5]. 

Adolescents and young adults (emerging adults) who use cannabis have a decline in the brain white matter integrity and the frontolimbic region volume. This results in more depressive symptoms compared to impulsivity [5].

Cannabis Use and Depression

Female emerging adults (those aged 18-25 years old) are one at-risk group for cannabis use disorder and clinical depression [6]. During this period, the use of cannabis peaks, and depression is the most common psychiatric disorder among emerging adults, with females having the greatest risk for depression [6]. One study compared occasional cannabis use to regular cannabis use. Occasional cannabis use is defined as using cannabis one to four times per week, while regular cannabis use is defined as using cannabis more than once per week [7]. The study illustrated that female who reported occasional cannabis use had higher levels of psychological distress than their male counterpart. Moreover, females who reported regular cannabis use had higher levels of psychological distress and were more likely to report experiencing suicidal thoughts and attempts. However, both females and males reported an increased odds ratio of major depressive episodes with occasional and regular cannabis use [7].

Another study evaluated how effective the association between depression and cannabis use disorder symptoms is based on the frequency of use, ranging from one day per month to daily use. The findings illustrated that the frequency of cannabis use has a strong association with cannabis use disorder [8]. However, an independent association was found for depressive symptoms when controlled for cannabis use frequency, age of onset of cannabis use, past month frequency of smoking, past month frequency of alcohol use, and past year of illicit substance use. The results suggested that adults with depression who use cannabis were more likely to communicate longer time usage and have difficulty cutting down. Moreover, they were more likely to communicate that cannabis usage affected important activities and affected their health. Lastly, they were more likely to develop tolerance than adults without depression who use cannabis [8].

Chronic cannabis use causes a reduction in the slow-wave sleep associated with daytime sleepiness, which often reduces the quality of sleep [9]. Chronic cannabis use is linked to both depressive disorder and subclinical depressive symptoms in adolescents and adults. Since the ECS regulates mood and the CNR 1, the code for the human CB 1 receptor and FAAH gene associated with mood disorders in humans, it is suggested that the ECS regulates mood, which has been associated with sleep problems [9].

Individuals who use cannabis and have depression are at a greater risk of having worse symptoms and functional outcomes [10]. Bahorik et al. compared the comorbidity of depression between medical and non-medical cannabis users. The study found that individuals using non-medical cannabis were more likely to present with suicidal ideation, depressive symptoms, and poorer mental health performance than individuals who did not use cannabis at baseline. Moreover, individuals using medical cannabis had poorer medical and physical health performance than individuals who did not use cannabis at baseline [10].

A recent study illustrated the temporal changes between cannabis use, suicidal ideation, and major depression between the periods of 2002 to 2012. This study suggested that at least monthly use of non-medical cannabis was associated with increased odds of major depressive episodes and suicidal ideation and both associations strengthened in 2012 as opposed to 2002 [11]. This strengthened association could be due to changes in the biochemical composition of cannabis, which has changed over time particularly in the illicit market; as such, the potency of delta-9-tetrahydrocannabinol (THC), the primary psychoactive cannabinoid in non-medical cannabis, has increased while cannabidiol (CBD) has decreased. It has been established that the prevalence of cannabis use among emerging adults is much higher than that in any other age groups with females at a greater risk of reporting co-occurring suicidal ideation. Since the brain is not fully developed until age 25 years, THC may disrupt normal neuronal development [11].

Another study illustrated the temporal changes between cannabis use, suicidal ideation, depression, and psychosis. This result is commonly seen in emerging adults aged 18-25 years who consume non-medical cannabis, particularly the delta-8-THC component. The delta-8-THC component has more synthetic properties, and affected individuals usually present with treatment-resistant depression and suicidal ideation. These individuals are usually treated with electroconvulsive therapy [12].

To further strengthen the above study, a retrospective cohort study was conducted to understand the relationship among MDD, suicidal thoughts, suicidal behavior, and cannabis involvement in discordant twins [13]. One study of monozygotic twins reared together who are discordant in cannabis use illustrated that the cannabis-dependent twin was at three to four conditional odds to report suicidal ideation and attempts even after adjusting for predisposing factors shared by twin pairs. Another study illustrated that the prevalence of MDD and suicidal ideation was greater in concordant exposed twins than in concordant unexposed twins [13].

Treatment

Even though research on the treatment of concurrent disorder is insufficient, it is vital to note that cognitive behavioral therapy is indicated for the treatment of both depression and cannabis-related problems along with antidepressant medication [11,13]. Additional treatment provided to engage the patient and improve adherence included substance use counseling, substance use resources, and a grief support group.

## Conclusions

Cannabis use disorder and clinical depression are prevalent in adults aged 18-25 years. Females in this age group are more likely to be at risk. In this case report, it is illustrated that the THC component in cannabis contributed to the neuropsychiatric decline in the 21-year-old female with a history of untreated depression, the death of her one-week-old son six months ago, and the recent death of her close aunt three weeks ago. With the cessation of cannabis over six days of hospitalization, her mental status slowly improved toward baseline. The ECS CB1 receptor slowly increased with the cessation of THC, which led to improvement in the patient's mood, sleep, and neuropsychiatric function. Although research on the treatment of concurrent disorders is insufficient, the current treatment regimen for concurrent depression and cannabis-related problems is cognitive behavioral therapy and antidepressant medications.
